# Common patterns of morbidity and multi-morbidity and their impact on health-related quality of life: evidence from a national survey

**DOI:** 10.1007/s11136-014-0820-7

**Published:** 2014-10-26

**Authors:** R. E. Mujica-Mota, M. Roberts, G. Abel, M. Elliott, G. Lyratzopoulos, M. Roland, J. Campbell

**Affiliations:** 1 Institute of Health Research, University of Exeter Medical School, Salmon Pool Lane, Exeter, EX2 4SG UK; 2 University of Plymouth Peninsula Schools of Medicine and Dentistry, Plymouth, UK; 3 Cambridge Centre for Health Services Research, University of Cambridge, Cambridge, CB2 0SR UK; 4 RAND, Santa Monica, CA USA; 5 Primary Care Research Group, University of Exeter Medical School, Exeter, EX1 2LU UK

**Keywords:** Quality of life, Equation 5D, Multi-morbidity

## Abstract

**Background:**

There is limited evidence about the impact of specific patterns of multi-morbidity on health-related quality of life (HRQoL) from large samples of adult subjects.

**Methods:**

We used data from the English General Practice Patient Survey 2011–2012. We defined multi-morbidity as the presence of two or more of 12 self-reported conditions or another (unspecified) long-term health problem. We investigated differences in HRQoL (EQ-5D scores) associated with combinations of these conditions after adjusting for age, gender, ethnicity, socio-economic deprivation and the presence of a recent illness or injury. Analyses were based on 831,537 responses from patients aged 18 years or older in 8,254 primary care practices in England.

**Results:**

Of respondents, 23 % reported two or more chronic conditions (ranging from 7 % of those under 45 years of age to 51 % of those 65 years or older). Multi-morbidity was more common among women, White individuals and respondents from socio-economically deprived areas. Neurological problems, mental health problems, arthritis and long-term back problem were associated with the greatest HRQoL deficits. The presence of three or more conditions was commonly associated with greater reduction in quality of life than that implied by the sum of the differences associated with the individual conditions. The decline in quality of life associated with an additional condition in people with two and three physical conditions was less for older people than for younger people. Multi-morbidity was associated with a substantially worse HRQoL in diabetes than in other long-term conditions. With the exception of neurological conditions, the presence of a comorbid mental health problem had a more adverse effect on HRQoL than any single comorbid physical condition.

**Conclusion:**

Patients with multi-morbid diabetes, arthritis, neurological, or long-term mental health problems have significantly lower quality of life than other people. People with long-term health conditions require integrated mental and physical healthcare services.

**Electronic supplementary material:**

The online version of this article (doi:10.1007/s11136-014-0820-7) contains supplementary material, which is available to authorized users.

## Introduction

In adults, the chronic diseases anxiety, depression, arthritis of the hip and knee and neurological conditions such as Parkinson’s disease have the largest negative association with health-related quality of life (HRQoL), while arthritis of the hip or knee, depression and back problems have the greatest burden at population level [1, 2, 3]. The magnitudes of these associations are consistent across countries despite differences in the prevalence of individual conditions [4]. Depression is the condition most strongly associated with HRQoL [2], resource use and costs [5], and its prevalence and evolution in an individual are associated with the presence of multiple chronic conditions [5–7]. Individuals with coexisting physical and mental health problems report the lowest HRQoL [8–10].

Currently, 18 to 30 % of adults live with two or more coexisting conditions [4, 9, 11] and more will do so as populations age. The implications of this trend for population HRQoL are unclear due to limited comparative evidence on the effects of multi-morbidity in young and old adults. Studies of multi-morbidity focus on the elderly [12–14] and the effects of specific combinations of chronic conditions [14, 15]. While there is evidence of synergistic effects for some combinations [15, 16], studies have lacked sufficient size to investigate the effects of specific, unselected, representative combinations [2, 10, 15–19] on HRQoL.

We aimed to estimate the associations of self-reported medical conditions, and specific combinations thereof, with HRQoL among respondents to a national survey of adults registered with English primary care practices.

## Methods

### Sample

We used data from the 2011–2012 English General Practice Patient Survey (GPPS), an annual national postal survey of adult patients' experience with primary care. Patients were randomly sampled with stratification [20] from the contact records of all GP practices across England. Questionnaires were sent out in two waves (July 2011 and January 2012), to around 1.4 and 1.36 million patients, respectively. Non-respondents were sent reminders in each of the 2 months following the initial mailings. Respondents were asked about the presence of any long-standing health conditions and invited to identify them from a list of twelve common conditions (angina or long-term heart problem, arthritis or long-term joint problem, asthma or long-term chest problem, cancer in the last 5 years, deafness or severe hearing impairment, diabetes, epilepsy, high blood pressure, kidney or liver disease, long-term back problem, long-term mental health problem and long-term neurological problem) or ‘another’ long-term condition [21]. The definition and self-reporting of long-term conditions was left to the respondent’s discretion.

### Measures

The survey incorporated the EuroQoL-5 dimensions (EQ-5D) classification system (but not EQ-5D VAS). The EQ-5D is a generic summary utility score of health state derived from individuals’ responses, on a three-level ordinal scale (no problems, moderate problems and severe problems), to each of its five dimensions (mobility, self-care, usual activities, pain/discomfort and anxiety/depression) [22]. We used UK tariffs [23] to obtain an index score (range −0.59, worst possible health state to 1, best possible health state). By construction, the value of 0 is equal to death and negative values represent HRQoL worse than being dead.

### Analysis

Initially, we analysed the joint occurrence of two conditions by estimating age-adjusted tetrachoric correlations of all 66 pairs of conditions, which adjust for differences in condition prevalence [24]. The relationship between EQ-5D and the number of long-term conditions was explored by age (≥65 vs. other) and mental health problem (yes vs. no) subgroups using nonparametric, local polynomial regressions [25].

We developed linear fixed-effects regression models of individual EQ-5D scores to estimate the associations of chronic conditions with HRQoL, whether alone or combined as comorbidities. Each model contained categorical indicators of the twelve specific conditions, ‘another long-term condition’, age (18–24 years, 25–84 in 10-year bands, and 85 and older), gender, ethnicity (mixed, Chinese, non-Chinese Asian, Black, Arab or any other ethnicity, and White), activity limitation by recent injury or affliction (yes, no), and a covariate for the index of multiple deprivation (expressed as a deviation from the sample mean) associated with respondents’ postcodes [26]. Interactions between age and each chronic condition and between the presence of a long-term mental health problem and each of the other chronic conditions were included. Reference categories were female gender, White ethnicity, age 55–64, no long-term condition and no recent injury affecting activity so that the constant represented the score for a person with these characteristics (and mean deprivation index). The models also included control variables indicating the presence of ‘Alzheimer’s disease or dementia’, ‘blindness or severe visual impairment’ and ‘learning difficulty’.

We estimated two models, which differed in how long-term conditions were measured as covariates of EQ-5D scores. The first ‘reductionist’ model included three categorical condition count variables indicating the presence of two, three, or four or more conditions, together with categorical indicators for each of the 11 physical conditions listed above, long-term mental health problems, and interactions between the latter and each physical condition. In the second ‘expanded’ model, instead of the condition count variables for two and three conditions, we estimated the adjusted interaction effects of all 66 condition pairs and 220 condition triplets that could be formed by the 12 conditions listed above. Since some specific combinations of three conditions had few available cases, we included all triplets in the model but reported only those with at least 20 observations. The EQ-5D score was the dependent variable in both models, but results for the expanded model were rescaled to full health equivalent days per year for ease of presentation.

We estimated the number of full health equivalent days lost per year associated with each condition by multiplying the relevant model (utility) coefficient by 365. We adopted the utility difference value of 0.03, i.e. 11 days of optimal health per year, as the minimally important difference across conditions [27, 28] and focused on estimated differences where the 95 % confidence interval only included values above 11 days.

In sensitivity analyses, we used a revised outcome measure that excluded the dimension of anxiety/depression from the utility score. Additionally, we estimated fixed-effects models that adjusted for selective survey non-participation [29] and item non-response [30]. Since these models involve untestable assumptions about the statistical distribution of non-response and non-participation, they are considered as secondary. All analyses incorporated person-level weights to account for practice stratification and post-stratification from differential non-participation by age, gender, ethnicity and neighbourhood deprivation (postcode) [31] in estimated coefficients and standard errors [32].

## Results

A total of 1,037,946 people (38 %) returned questionnaires including 44,808 completed online and 64 by phone, with 1,012,976 respondents completing at least one EQ-5D question. Prevalence data are reported for 906,578 respondents, excluding 131,368 (12.7 %) who did not say whether they had a long-term condition. Analyses of EQ-5D scores are limited to 831,537 respondents (80.1 %) after excluding those who had incomplete answers to EQ-5D (74,467) or missing information on deprivation (574). Appendix Table A1 shows respondents’ socio-demographic characteristics.

Overall, 53.2 % of respondents reported at least one long-term health problem (Table 1), the most common being high blood pressure (18.7 %), arthritis or long-term joint problems (13.4 %), asthma or long-term chest problems (10.8 %), and long-term back problems (10.3 %). Prevalence estimates from our sample were comparable with similar population measures from the British Household Panel Study, Quality and Outcomes Framework or General Household Survey (Appendix Table A2), except for diabetes, high blood pressure and back problems, where our GPPS-derived estimates were 33–60 % higher than those from other sources.

**Table 1:** Prevalence of medical conditions and EQ-5D health state (906,578 respondents unless stated otherwise)

	Number of respondents	Weighted percentage^a^	EQ-5D scores mean
Medical condition			
Angina or long-term heart problem	66,971	5.3	0.63
Arthritis or long-term joint problem	166,981	13.4	0.54
Asthma or long-term chest problem	102,070	10.8	0.74
Cancer in last 5 years	37,433	3.0	0.70
Deafness or severe hearing impairment	49,017	4.0	0.63
Diabetes	85,760	7.1	0.69
Epilepsy	10,873	1.3	0.67
High blood pressure	233,124	18.7	0.73
Liver or kidney disease	17,903	1.6	0.59
Long-term back problem	108,968	10.3	0.53
Long-term mental health problem	35,397	4.3	0.50
Long-term neurological problem	18,448	1.8	0.44
Another long-term condition	118,251	12.0	0.68
None of these conditions	314,659	46.8	0.94
Pattern of morbidity/comorbidity			
No. of medical conditions, mean (SD)	906,578	0.94 (1.6)	
One condition	302,117	31.3	0.81
Two or more conditions	275,458	23.3	0.60
Two conditions	152,932	13.5	0.69
Three conditions	73,260	5.9	0.54
Four conditions	31,618	2.5	0.41
Five conditions	11,795	0.9	0.31
Six or more conditions	5,853	0.5	0.19
EQ-5D score mean (SD)	831,537		0.82 (0.26)
EQ-5D domains			
No problems in any EQ-5D domains	369,662	50.1	1.00
Moderate problem in ≥1 EQ-5D domains	457,462	49.3	0.65
Severe problem in ≥1 EQ-5D domains	77,846	8.1	0.10
Severe problem in all EQ-5D domains	222	0.0	−0.59

^a^Weights accounted for stratification of the sample at practice level and for variable probability of survey non-participation by age, gender, and ethnicity composition and neighbourhood deprivation [31]

**Fig. 1:**
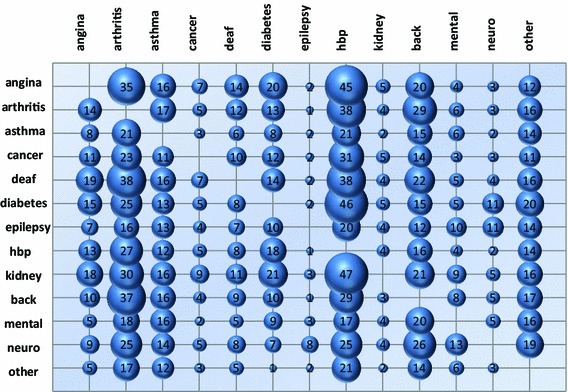
Comorbidity prevalence (%) by medical condition index conditions (*rows*), comorbidities (*columns*). The area of each bubble is proportional to the percentage of respondents with the condition identified by the row label who also reported the comorbidity identified in the columns. The range of comorbidity prevalence runs from 47 % for high blood pressure in respondents with kidney/liver disease to 1 % for epilepsy in respondents with high blood pressure. *Notes*: angina = angina or long-term heart problem, arthritis = arthritis or long-term joint problem, asthma = asthma or long-term chest problem, cancer = cancer in the last five years, deaf = deafness or severe hearing impairment, hbp = high blood pressure, kidney = kidney or liver disease, back = long-term back problem, mental = long-term mental health problem, neuro = long-term neurological problem, other = another long-term problem

Multi-morbidity, the presence of two or more long-term conditions, was reported by 23 % of all respondents, varying from 7 % of those under 45 years of age to 51 % of those 65 years and older. Figure 1 shows the prevalence of combinations of two conditions. As expected, the most prevalent combinations include common conditions such as high blood pressure and angina. Five pairs of conditions had an age-adjusted tetrachoric correlation ≥0.25 (none was ≤−0.25): arthritis and back problems, 0.43; epilepsy and long-term neurological problems, 0.36; diabetes and hypertension, 0.28; kidney or liver disease and hypertension, 0.27; and long-term neurological and mental health problems, 0.25.

**Fig. 2:**
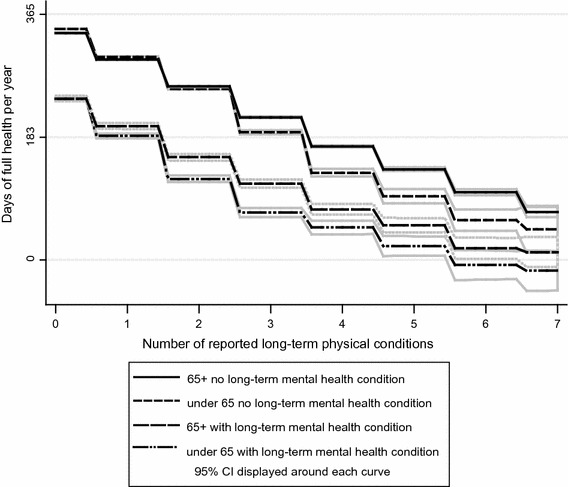
Quality of life score (EQ-5D × 365) declines with increasing morbidity count. Unadjusted nonparametric regression of EQ-5D scores against number of long-term physical conditions, separately for each of the four subgroups formed by age (under 65 vs. older) and the presence of mental health condition (yes vs. no) categories

Health status varied substantially; 50 % of respondents reported no problems in any EQ-5D dimension, whereas 0.02 % reported severe problems in all dimensions (Table 1). Compared to data from the Health Survey for England (HSE) 2008, the most recently available on national norms (Appendix Table A3), the analysis sample had a lower mean EQ-5D score (0.82 vs. 0.86; difference: 0.04 × 365 = 14 days). Older and younger respondents were similar with respect to the association of chronic disease and HRQoL for up to two physical conditions. However, having more than two physical conditions was associated with less reduction in HRQoL for older respondents than for younger respondents. Long-term mental health problems were also associated with smaller EQ-5D differences among older than younger respondents (Fig. 2). These patterns were unaffected by omitting the anxiety/depression dimension from the EQ-5D score (results available from the authors).

**Fig. 3:**
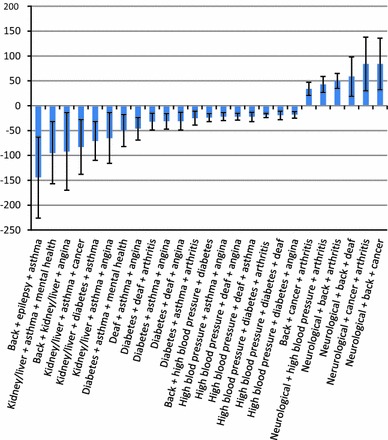
Three condition combinations with the largest interaction effects (depicted with 95 % CI *bars*) on full health equivalent days per year (EQ-5D × 365), GPPS 2011–12. *Note*: Out of 220 triplet combinations, these are 25 whose 95 % CI did not include any of the values in the range [−11, 11]. The full list of combinations and their interaction effects is in Appendix, Table A5. Model 2 with dyads & triads versus dyads only *F* = 19.3 (286, 823121), *p* < 0.0001. For a description of the labels, see footnote to Fig. 1

**Fig. 4:**
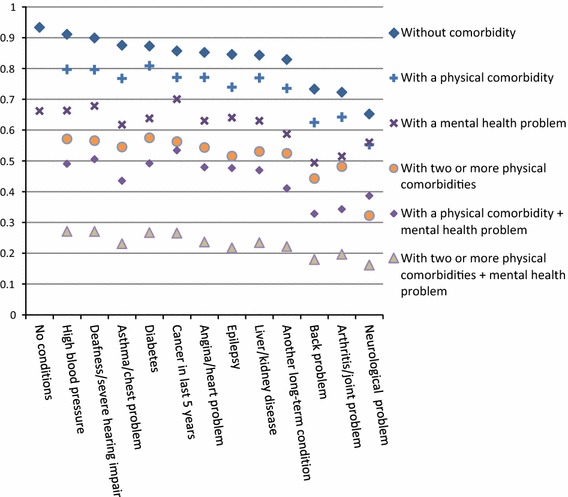
Adjusted mean utility scores of 55–64-year-old White woman of average level of material deprivation, by chronic condition. Based on predictions from Model 1, for a White, female, age 55–64 person of mean socioeconomic level, using a linear fixed-effects model adjusting for age, gender, ethnicity, index of multiple deprivation, recent illness or injury, and interactions of age and chronic conditions (see Appendix Tables A4 and A5 for detailed analyses of the impact of combinations of conditions on quality of life). For a description of condition labels, see footnote to Fig. 1

 For individual conditions, long-term neurological problems and long-term mental health problems were associated with the greatest reduction in HRQoL, equivalent to 99 and 78 days, respectively (Table 2). For some combinations of two or three conditions, the combined difference associated with multiple conditions was less than the sum of the individual condition coefficients (‘sub-additive’ interactions) or more than the sum of the individual condition coefficients (‘super-additive’ interactions): 21 super-additive and 13 sub-additive combinations were clinically meaningful. Of these, only two super-additive and seven sub-additive combinations were found among paired conditions (Table 2); the super-additive combinations were diabetes with back problems and diabetes with arthritis. Stronger associations were found for triplet combinations (Fig. 3, Appendix Table A5), where the largest synergistic, super-additive associations were for long-term back problems with epilepsy and asthma or chest problems (144 additional full health-equivalent days of life lost, 95 % CI 63–226) and long-term mental health with asthma or chest problems and liver or kidney disease (95, 95 % CI 32–157). The condition appearing most frequently in clinically significant super-additive triplets was asthma (most often in combination with angina or heart problem), followed by diabetes (most often in combination with hypertension). Figure 4 summarises the effects of additional comorbidities predicted by the ‘reductionist’ model. This shows that, for people with one or two physical problems, the addition of a mental health problem was associated with a greater impact on HRQoL than the addition of a second or third physical condition.

**Table 2:** Interaction effects on full health equivalent days per year (EQ 5D × 365) for all combinations of two long-term conditions, GPPS 2011–12 (95 % CI)

	Angina/heart	Arthritis/joint	Asthma	Cancer	Deaf/severe hearing	Diabetes	Epilepsy	High blood pressure	Kidney/liver	Back	Mental health	Neurological
Angina/heart	−25 (−28, −23)											
Arthritis/joint	3 (−2, 8)	−70 (−72, −69)										
Asthma	*−15 (21, −9)*	−1 (−5, 3)	−16 (−18, −14)									
Cancer	*10 (3, 18)*	***20 (13, 26)***	4 (−3, 11)	−40 (−43, −38)								
Deaf/severe hearing	*−8 (−15, −1)*	−2 (−8, 3)	*−15 (−22, −8)*	4 (−5, 12)	−17 (−20, −13)							
Diabetes	−4 (−8, 1)	***−16 (−22,−11)***	*−11 (−18, −4)*	−7 (−18, 3)	*−7 (−14, −1)*	−13 (−15, −11)						
Epilepsy	2 (−18, 22)	−6 (−21, 10)	−14 (−29, 0)	−18 (−46, 9)	−16 (−37, 5)	−19 (−42, 4)	−10 (−16, −4)					
High blood pressure	*−6 (−9,−3)*	−8 (−10, 5)	*−3 (−6, −1)*	*9 (5, 12)*	*−10 (−13, −6)*	*−4 (−7, −2)*	−8 (−18, 2)	−7 (−8, −6)				
Kidney/liver	0 (−25, 26)	10 (−3, 23)	−18 (−38, 1)	*14 (0, 28)*	10 (−13, 34)	*−18 (−31, −6)*	−10 (−47, 27)	*7 (0,14)*	−34 (−39, −28)			
Back	3 (−5, 11)	***23 (19, 27)***	1 (−4, 5)	***24 (16, 32)***	*8 (0, 15)*	***−24 (−34, −15)***	−23 (−51, 4)	*−5 (−9, −1)*	12 (−3, 27)	−64 (−66, −61)		
Mental health	−13 (−39,12)	1 (−9,11)	*−9 (−18, −1)*	12 (−6, 29)	−9 (−26, 9)	−8 (−21, 4)	*−35 (−62, −7)*	*−9 (−18, −1)*	−11 (−36,14)	−7 (−15, 2)	−78 (−82, −74)	
Neurological	11 (−15, 38)	***34 (17, 51)***	*19 (1, 37)*	*32 (9, 54)*	***42 (17, 68)***	−18 (−42, 6)	11 (−12, 34)	*14 (3, 24)*	21 (−32, 74)	***36 (22, 51)***	***56 (35, 76)***	−99 (−105, −93)

Italicised cells are those of statistically significant interactions at the 5 % level. Figures in bold italics are estimated interactions with 95 % CI that did not include values in the interval [−11, 11]; i.e. clinically significant interactions. Model 2 with dyads only versus no interactions, *F* = 19.3 (286, 823121), *p* < 0.0001

Similar relative magnitudes of these associations were found in the analyses that excluded responses on the EQ-5D anxiety/depression dimension from the index score (results available from the authors). Correcting for informative EQ-5D item non-response resulted in negligible differences in the results (Appendix, Table A4), and survey non-participation was non-informative (results available from the authors).

## Discussion

We estimated the strength of associations of chronic conditions with HRQoL in a large national survey of English NHS patients. The large sample of self-reported morbidity across 13 conditions (including a non-specified long-term condition) allowed us to estimate interaction effects of coexisting conditions with low prevalence not observable in smaller studies [2, 16, 17]. Neurological disease, which has low prevalence, arthritis and long-term back problems were the physical conditions most strongly associated with reduced HRQoL. Excluding neurological conditions, the presence of a comorbid mental health problem had a greater adverse effect on HRQoL than any single comorbid physical condition. The association of long-term medical problems and lower HRQoL was stronger in younger adults, especially those with multiple comorbidities.

Consistent with international [33] and US [34] reports, mental health, arthritis, joint and back problems had the largest effects on HRQoL among common chronic conditions. Contrary to previous studies using the EQ-5D [35] or SF-6D [10], we found increasing marginal disutility within the 0–3 range of the multi-morbidity count. We also found 16 pairs and 35 triplets of coexisting long-term conditions with negative interaction effects on HRQoL, of which 2 and 19, respectively, were sufficiently large and precisely estimated to make a difference on a person’s life. There is, however, no previous assessment of a comprehensive set of combinations of conditions in a national adult sample with which to compare our results.

The association between multi-morbidity and reduced HRQoL may reflect several factors. Super-additive associations may reflect clinically synergistic effects, as in the case of diabetes and hypertension, whose coexistence may accelerate atherosclerotic coronary, cerebrovascular, and peripheral vascular disease; may result from one condition hampering the patient’s ability to adhere to treatment for another, as in the case of osteoarthritis occurring in diabetics [36]; or may occur with conditions affecting different systems and HRQoL dimensions, such as hearing impairment and long-term back problems. The most prevalent sub-additive association was the dyad of arthritis/joint and long-term back problem, which are likely to have a common aetiology and manifest primarily through pain and mobility limitations.

Our analysis had limitations. Respondents’ educational level was not recorded in the survey, and age was recorded in ten-year bands. Our HRQoL measure (EQ-5D) has limited ability to discriminate the effect of mild medical conditions [37] and cognitive [38] or sensory impairments [39].

The analysis may be affected by the failure of self-reported conditions to correspond with objective health status characteristics [40–42], under-reporting of conditions, including comorbid depression [43], and by survey non-participation and item non-response [44]. Women, the middle aged and those in affluent areas are more likely to respond to postal surveys [45]. Prevalence of individual long-term conditions in our sample was similar to comparable reports from other English sources, except diabetes, high blood pressure and long-term back problems. Besides over-representation of these conditions in our sample, possible explanations for the differences include receding reference surveys, and the framing effects of wording or context differences in relevant questions.

The average GPPS respondent had two fewer weeks of full health per year than the most recent available norm, from HSE. While this difference may reflect the increased participation of less healthy, more frequent users of healthcare services in the GPPS [46] relative to the HSE population, it is unlikely to affect the results of our analyses, which adjusted for age, gender, ethnicity, and deprivation and, to the extent the issue could be assessed, survey non-participation and informative item non-response.

The weaker association of medical conditions with HRQoL among older people does not appear wholly attributable to floor effects since the age < 65 and age ≥ 65 groups have similar ‘baseline’ EQ-5D scores in the absence of any long-term condition and may reflect lower expectations of health in older age or a greater ability to adapt to lifestyle changes imposed by adverse health events. Results of further analyses excluding the responses to the anxiety/depression dimension from the EQ-5D score confirmed these findings.

Our results also highlight the association of certain physical conditions with reduced HRQoL, in particular diabetes, especially when occurring alongside other conditions. We found that people with diabetes and arthritis or joint problem or a long-term back problem have disproportionately larger HRQoL deficits than those experienced by individuals with only one of these conditions, relative to otherwise similar persons with no reported condition. Since individual reporting of these conditions is not correlated, there is the risk that patients, their general practitioners and the specialists treat each condition as independent from one another, thus missing the opportunity to address the impairment on HRQoL that is uniquely associated with the interaction of the conditions. Similar observations apply to mental health problems, which are a substantial cause of morbidity in their own right, but also associated with substantial HRQoL deficits when coexisting with multiple physical conditions, e.g. cardiovascular complications of diabetes due to poor treatment adherence and glycaemic control [47–49]. Although the associations of mental health with HRQoL presented here may reflect causal effects in both directions, the associations of physical health with HRQoL were stronger in the presence of long-term mental health problems. This highlights the importance of addressing mental health issues in patients who may be attending multiple clinics for their physical problems while their mental wellbeing is overlooked. Our findings add weight to calls [9, 50] for integrated approaches to the diagnosis and treatment of long-term health conditions.

## Electronic supplementary material

Below is the link to the electronic supplementary material.

Supplementary material 1 (DOCX 92 kb)
