# Supplementary material for: Common patterns of morbidity and multi-morbidity and their impact on health-related quality of life: evidence from a national survey
Source: Qual Life Res. 2014 Oct 26;24(4):909–18. doi: 10.1007/s11136-014-0820-7 (PMC4366552; doi:10.1007/s11136-014-0820-7)
Supplement: Supplementary file 1 — Supplementary material 1 (DOCX 92 kb) [file 11136_2014_820_MOESM1_ESM.docx]

**Appendix**

Appendix Table A1 Sample characteristics (N= 831, 537 adults aged >18 years)

|  |  | GPPS  (N=1037,946) | | Analysis sample  (N=831,537*) | |
| --- | --- | --- | --- | --- | --- |
| Personal characteristics of respondent |  | N | Weighted Statistic (Proportion, except where indicated by^) | N | Weighted Statistic (Proportion, except where indicated by^) |
| Age | 18-34 years | 1,017,964 | 0.27 | 820,040 | 0.27 |
|  | 35-64 years | 1,017,964 | 0.52 | 820,040 | 0.52 |
|  | older than 64 years | 1,017,964 | 0.21 | 820,040 | 0.21 |
| Gender | Male | 1,017,860 | 0.49 | 820,174 | 0.49 |
| Religion: | None | 981,657 | 0.26 | 795,527 | 0.26 |
|  | Christian | 981,657 | 0.64 | 795,527 | 0.64 |
|  | Other religion | 981,657 | 0.10 | 795,527 | 0.09 |
| Smoker: | Regular | 1,002,639 | 0.11 | 812,218 | 0.11 |
| Support family due to their ill health |  | 959,997 | 0.18 | 787,432 | 0.18 |
| Number of chronic conditions (out of 13) | Any | 906,578 | 0.55 | 831,537 | 0.54 |
|  | Mean number (SD) | 906,578 | 0.94 (1.16) | 831,537 | 0.90^ (1.13^) |
| Ethnicity | White | 1,016,835 | 0.87 | 818,840 | 0.88 |
|  | Mixed race | 1,016,835 | 0.01 | 818,840 | 0.01 |
|  | Asian | 1,016,835 | 0.06 | 818,840 | 0.06 |
|  | Chinese | 1,016,835 | 0.01 | 818,840 | 0.01 |
|  | Black African/ Caribbean | 1,016,835 | 0.03 | 818,840 | 0.02 |
|  | Other ethnicity/race | 1,016,835 | 0.03 | 818,840 | 0.02 |
| Socio-economic deprivation | Mean IMD score (SD) | 1,037,226 | 21.90 (15.59^) | 831,537 | 21.46^ (15.39^) |
| Employment status | Full Time work | 976,211 | 0.43 | 792,441 | 0.44 |
|  | Part-time work | 976,211 | 0.14 | 792,441 | 0.13 |
|  | Full-time education | 976,211 | 0.03 | 792,441 | 0.03 |
|  | Unemployed | 976,211 | 0.06 | 792,441 | 0.05 |
|  | Retired | 976,211 | 0.21 | 792,441 | 0.21 |
|  | Permanently sick/disabled | 976,211 | 0.05 | 792,441 | 0.05 |
|  | Looking after the home or doing something else | 976,211 | 0.08 | 792,441 | 0.08 |
| Sexual orientation | Hetero-sexual/ straight | 972,652 | 0.93 | 796,073 | 0.94 |
|  | Other | 972,652 | 0.02 | 796,073 | 0.02 |
|  | Prefer not to say | 972,652 | 0.05 | 796,073 | 0.04 |
| Setting | Rural location of GP practice** | 1,037,946 | 0.15 | 831,537 | 0.15 |

*Missing data (%) in the analysis sample (defined on the basis of availability of data on EQ-5D index score, long term conditions, and socio-economic deprivation based on postcode of respondent’s home): age, 1.38; gender, 1.37; religion, 4.37; smoking status: 2.32; looks after family, 5.30; any condition, 0 (sample inclusion criterion); race, 1.53; socioeconomic deprivation, 0 (sample inclusion criterion ); employment status, 4.93; sexual orientation, 4.26; rural residence, 0. ** In three GP practices, respondents with a recorded urban location of practice and respondents with a recorded rural practice location were assigned to the same practice. This might reflect practice relocation during the survey data collection period. Respondents from these practices were all coded as having an urban practice, resulting in recoding of data for n=193 respondents from a rural to an urban practice location category.

Appendix Table A2. Comparison between prevalence of common conditions in the GP Patient Survey and other estimates of condition prevalence

|  | Number of respondents | Weighted percentage in GP Patient Survey * | Estimates of condition prevalence from other sources |
| --- | --- | --- | --- |
|  |  |  |  |
| Medical condition |  |  |  |
| Angina or long term heart problem | 66,971 | 5.3 | 3.4^1^, 5.3^2^ |
| Arthritis or long term joint problem | 166,981 | 13.4 | 15.6^3^ |
| Asthma or long term chest problem | 102,070 | 10.8 | 7.5^4^, 10.4^5^, 14.3^6^ |
| Cancer in last 5 years | 37,433 | 3.0 | 1.8^7^, 1.7^8^, 3.3^9^ |
| Deafness or severe hearing impairment | 49,017 | 4.0 | 4.0^10^,9.0^11^ |
| Diabetes | 85,760 | 7.1 | 5.5^12^, 5.6^13^ |
| Epilepsy | 10,873 | 1.3 | 0.8^12^, 0.8^13^ |
| High blood pressure | 233,124 | 18.7 | 14.3^14^ |
| Liver or kidney disease | 17,903 | 1.6 | 4.3^15^; 1.0- 1.3^16^ |
| Long term back problem | 108,968 | 10.3 | 6.0^17^ |
| Long term mental health problem | 35,397 | 4.3 | ^18^ |
| Long term neurological problem | 18,448 | 1.8 | N/A |

| Notes  * Weights account for stratification of the sample at practice level, and for probability of survey nonparticipation by age, gender, and ethnicity composition and neighbourhood deprivation.  1. 3.4% is the prevalence of coronary heart disease from the Quality and Outcomes Framework, whereas the figure of 5.2% from the GP Patient Survey will include other heart conditions. QOF data also gives prevalence for heart failure of 0.7% and atrial fibrillation of 1.4%, but these cannot be added to the QOF prevalence of 3.4% for CHD as many cases of heart failure and atrial fibrillation will also have CHD. |
| --- |
| 2. Estimate of 5.3% for CHD from British Heart Foundation www.bhf.org.uk/publications/view-publication.aspx?ps=1001546, based on data in the Health Survey for England. |
| 3. From: A Heavy Burden: the occurrence and impact of musculoskeletal conditions in the United Kingdom today. Parsons et al, 2011, based on General Household Survey data. <http://www.medicine.manchester.ac.uk/musculoskeletal/aboutus/publications/heavyburden.pdf>  4. Combined prevalence of asthma (5.9%) and COPD (1.6%) from Quality and Outcomes Framework.  5. The more inclusive figure of 10.4% comes from adding the figure for current asthma from QOF (5.9%) to the 4.5% lifetime prevalence of doctor diagnosed "chronic bronchitis, emphysema, or COPD" from the Health Survey for England (2010)  6. British Household Panel Survey (2009) reports prevalence of 14.3% for "Chest/breathing problems, asthma, bronchitis", but without specifying 'long term'.  7. Cancer since 2003 from Quality and Outcomes Framework = 1.6% excluding skin cancer. Skin cancer prevalence estimated at 0.2% from CRUK figures of 37,321 malignant melanoma in last five years and 99,549 non-melanoma skin cancer (prevalence period unspecificed, likely to be more than five years but noted by CRUK to be a substantial underestimate). www.cancerresearchuk.org/cancer-info/cancerstats/types/skin/incidence/uk-skin-cancer-incidence-statistics#prev  8 British Household Panel Survey (2009) reports prevalence of 1.7% for 'cancer'. This question might be expected to give a lower prevalence than 'cancer in past five years'.  9. Figure of 3.3% is a 17 year prevalence estimate for cancers diagnosed from 1971 to 2008 for people alive in 2009 (Maddams et al. British Journal of Cancer 2102; 107: 1195-1207)  10. 4% of the population has >45dB hearing loss in both ears (from Davis A C. The prevalence of hearing impairment and reported hearing disability in adults in Great Britain. Int J Epidem 1989, 18: 911–7). Much higher percentages report more minor degrees of hearing loss, for example 21.1% of people in Scotland report that they find it 'very difficult' to follow a conversation if the background is noisy (Hannaford et al Family Practice 2005; 22: 277).  11. Questions on hearing impairment were asked with similar wording in previous rounds of the GP Patient Survey and produced higher figures (for 2008/09, 2009/10, 2010/11 respectively: deafness or severe hearing impairment 8.2%, 8.9%, 9.3%. In earlier years, the questions were asked in the context of 'disability' whereas in 2011/12 the questions were asked in terms of 'which of the following medical conditions do you have?'. The lower prevalence of positive response for this condition in 2011/12 may have been due to respondents not regarding it as 'medical condition' but may also relate to the position of the question in a much longer list of conditions with the instruction 'tick all that apply to you'. Previous research suggests that where a 'Yes/No' response is not required for each question, there may be under-reporting of less salient conditions. We also noted that in 2011/12, the majority of people who said that they were 'deaf and used sign language' did not endorse deafness as a medical condition.  12. Data from the Quality and Outcomes Framework www.qof.ic.nhs.uk/  13. British Household Panel Survey (2009) https://www.iser.essex.ac.uk/bhps  14. From Health Survey for England. Includes only those on treatment. http://www.ic.nhs.uk/statistics-and-data-collections/health-and-lifestyles-related-surveys/health-survey-for-england/health-survey-for-england--2009-trend-tables  15. 4.3% is the prevalence of 'Chronic kidney disease' from the Quality and Outcomes Framework. However, the QOF definition of this is based on a biochemical definition of reduced GFR and it is believed that many people included in this register would not regard themselves as having kidney disease.  16. 1.0% of men and 1.3% of women reported having doctor-diagnosed chronic kidney disease (CKD) in the Health Survey for England [HSE2009 and HSE2010 data combined]  17. Published population surveys of back pain generally report one or twelve months incidence or prevalence of back pain which cannot be easily related to the phrasing of 'long term back problem' in GPPS because of the episodic nature of back pain. The 6.0% estimate given here is based on a Department of Health Report in 1998 in which 40% of people reported that they had experienced back pain in the previous year and 15% of these reported that they were in pain throughout the year http://webarchive.nationalarchives.gov.uk/+/www.dh.gov.uk/en/Publicationsandstatistics/Publications/PublicationsStatistics/DH_4006687  18. In the 2009 GP Patient Survey, 5.7% of respondents reported a "longstanding psychological or emotional problem". The 2003 Health Survey for England (Department of Health 2003) reported a prevalence of people with a GHQ score of four or more was 15% in women and of 11% in men. The Adult Psychiatric Morbidity Survey (NHS Information Centre 2009) using the revised Clinical Interview Schedule found clinical significant neurotic symptoms in 17% in women and 12% in men. These last two surveys give higher estimates of psychiatric morbidity, but both estimated incident psychiatric symptoms which could have been short term, whereas the question in the GP Patient Survey, to which 4.2% of respondents responded positively, asked specifically about a long term mental health problem. |

Appendix Table A3. Comparison between EQ-5D scores and responses in the GP Patient Survey and Health Survey for England 2008

|  | Number of respondents reporting the outcome (N=831,537)* | Weighted estimate in GP Patient Survey | Weighted estimates in Health Survey for England 2008 |
| --- | --- | --- | --- |
|  |  |  |  |
| EQ_5D score mean (SD) | 831,537 | 0.82 (0.26) | 0.86 (0.23) |
| EQ-5D domains (%) |  |  |  |
| No problems in any EQ-5D domain domain | 369,662 | 50.1 | 55.2 |
| Moderate problem in ≥1 EQ-5D domains | 457,462 | 49.3 | 44.3 |
| Severe problem in ≥1 EQ-5D domains | 77,846 | 8.1 | 6.5 |
| Severe problem in all EQ-5D domains | 222 | 0.0 | 0.7 |

* Estimating sample after excluding respondents stating ‘prefer not to say to question about presence of specific long term conditions and those without data on multiple deprivation of postcode. See footnote to Appendix Table 1 for details.

Appendix Table A4. Linear models (weighted) of EQ-5D score and chronic conditions, and demographic and socio-economic characteristics

|  | **Fixed effects model**  **(N=831,537)** | | | **Self-selection model**  **(N=796,073)**** | | |
| --- | --- | --- | --- | --- | --- | --- |
| **Variable** | **Coefficient** | **95% CI**  **Lower, Upper** | | **Coefficient** | **95% CI**  **Lower, Upper** | |
| Angina/long-term heart problem | -0.081 | -0.087 | -0.075 | -0.075 | -0.081 | -0.070 |
| Arthritis/long-term joint problem | -0.211 | -0.215 | -0.206 | -0.194 | -0.197 | -0.190 |
| Asthma/long-term chest problem | -0.058 | -0.063 | -0.053 | -0.050 | -0.054 | -0.046 |
| Cancer in the last 5 years | -0.076 | -0.083 | -0.069 | -0.069 | -0.075 | -0.063 |
| Deafness/ severe hearing impairment | -0.034 | -0.042 | -0.026 | -0.027 | -0.034 | -0.020 |
| Diabetes | -0.060 | -0.065 | -0.056 | -0.056 | -0.060 | -0.052 |
| Epilepsy | -0.088 | -0.104 | -0.071 | -0.063 | -0.076 | -0.051 |
| High blood pressure | -0.022 | -0.025 | -0.019 | -0.022 | -0.025 | -0.019 |
| Kidney or liver disease | -0.090 | -0.102 | -0.078 | -0.077 | -0.088 | -0.066 |
| Long-term back problem | -0.200 | -0.205 | -0.195 | -0.185 | -0.189 | -0.180 |
| Long term mental health problem | -0.271 | -0.282 | -0.261 | -0.248 | -0.257 | -0.238 |
| Long-term neurological problem | -0.281 | -0.294 | -0.268 | -0.261 | -0.271 | -0.250 |
| Another long-term condition | -0.104 | -0.108 | -0.100 | -0.104 | -0.108 | -0.101 |
| Angina/long-term heart problem x MH | 0.049 | 0.024 | 0.074 | 0.040 | 0.012 | 0.067 |
| Arthritis or long-term joint problem x MH | 0.064 | 0.047 | 0.080 | 0.046 | 0.028 | 0.064 |
| Asthma or long-term chest problem x MH | 0.014 | -0.002 | 0.029 | 0.007 | -0.009 | 0.024 |
| Cancer in the last 5 years x MH | 0.115 | 0.083 | 0.146 | 0.132 | 0.095 | 0.169 |
| Deafness or severe hearing impairment x MH | 0.050 | 0.027 | 0.074 | 0.047 | 0.021 | 0.073 |
| Diabetes x MH | 0.037 | 0.018 | 0.055 | 0.042 | 0.021 | 0.063 |
| Epilepsy x MH | 0.066 | 0.029 | 0.103 | 0.083 | 0.041 | 0.125 |
| High blood pressure x MH | 0.024 | 0.008 | 0.039 | 0.022 | 0.005 | 0.039 |
| Kidney or liver disease x MH | 0.058 | 0.025 | 0.091 | 0.067 | 0.030 | 0.105 |
| Long-term back problem x MH | 0.032 | 0.016 | 0.048 | 0.019 | 0.002 | 0.037 |
| Long-term neurological problem x MH | 0.179 | 0.153 | 0.205 | 0.167 | 0.139 | 0.195 |
| Another long-term condition x MH | 0.029 | 0.014 | 0.045 | 0.026 | 0.009 | 0.043 |
| Indicator 2 conditions | 0.006 | 0.003 | 0.008 | 0.004 | 0.001 | 0.007 |
| Indicator 3 conditions | -0.004 | -0.009 | 0.001 | -0.001 | -0.007 | 0.004 |
| Indicator 4+ conditions | -0.012 | -0.020 | -0.003 | -0.005 | -0.013 | 0.003 |
| Indicator 2 conditions x MH | -0.099 | -0.120 | -0.078 | -0.103 | -0.125 | -0.080 |
| Indicator 3 conditions x MH | -0.125 | -0.157 | -0.094 | -0.128 | -0.162 | -0,094 |
| **Variable** | **Coefficient** | **95% CI**  **Lower, Upper** | | **Coefficient** | **95% CI**  **Lower, Upper** | |
| Indicator 4+ conditions x MH | -0.123 | -0.170 | -0.075 | -0.117 | -0.168 | -0.066 |
| Angina x Age 65-74 | 0.017 | 0.010 | 0.025 | 0.015 | 0.010 | 0.021 |
| Angina x Age 75-84 | 0.030 | 0.023 | 0.038 | 0.018 | 0.012 | 0.023 |
| Angina x Age 85+ | 0.047 | 0.036 | 0.058 | 0.025 | 0.016 | 0.034 |
| Arthritis x Age 65-74 | 0.021 | 0.016 | 0.026 | 0.013 | 0.009 | 0.017 |
| Arthritis x Age 75-84 | 0.041 | 0.035 | 0.046 | 0.018 | 0.013 | 0.022 |
| Arthritis x Age 85+ | 0.057 | 0.048 | 0.066 | 0.008 | -0.001 | 0.016 |
| Asthma/long term chest prob. x Age 65-74 | -0.007 | -0.013 | -0.000 | -0.001 | -0.006 | 0.004 |
| Asthma/long term chest prob. x Age 75-84 | -0.004 | -0.011 | 0.003 | -0.004 | -0.010 | 0.001 |
| Asthma/long term chest prob. x Age 85+ | 0.010 | -0.003 | 0.023 | 0.002 | -0.010 | 0.014 |
| Cancer in the last 5 years x Age 65-74 | 0.019 | 0.010 | 0.027 | 0.010 | 0.003 | 0.018 |
| Cancer in the last 5 years x Age 75-84 | 0.036 | 0.027 | 0.046 | 0.015 | 0.007 | 0.023 |
| Cancer in the last 5 years x Age 85+ | 0.055 | 0.040 | 0.070 | 0.023 | 0.011 | 0.035 |
| Deafness/severe hearing imp. x Age 65-74 | 0.015 | 0.006 | 0.025 | 0.007 | -0.000 | 0.015 |
| Deafness/severe hearing imp. x Age 75-84 | 0.016 | 0.006 | 0.025 | 0.009 | 0.002 | 0.017 |
| Deafness/severe hearing imp. x Age 85+ | -0.005 | -0.017 | 0.007 | -0.007 | -0.016 | 0.003 |
| Diabetes x Age 65-74 | 0.004 | -0.002 | 0.010 | 0.002 | -0.003 | 0.006 |
| Diabetes x Age 75-84 | 0.011 | 0.004 | 0.018 | 0.004 | -0.001 | 0.009 |
| Diabetes x Age 85+ | 0.039 | 0.027 | 0.051 | 0.021 | 0.010 | 0.032 |
| Epilepsy x Age 65-74 | 0.031 | 0.009 | 0.053 | 0.016 | 0.001 | 0.030 |
| Epilepsy x Age 75-84 | 0.032 | 0.007 | 0.057 | 0.020 | 0.001 | 0.038 |
| Epilepsy x Age 85+ | 0.019 | -0.024 | 0.062 | 0.016 | -0.015 | 0.048 |
| High blood pressure x Age 65-74 | 0.007 | 0.003 | 0.011 | 0.005 | 0.002 | 0.008 |
| High blood pressure x Age 75-84 | 0.015 | 0.011 | 0.020 | 0.009 | 0.005 | 0.012 |
| High blood pressure x Age 85+ | 0.039 | 0.031 | 0.047 | 0.021 | 0.014 | 0.027 |
| Kidney/liver disease x Age 65-74 | 0.008 | -0.008 | 0.024 | 0.004 | -0.010 | 0.018 |
| Kidney/liver disease x Age 75-84 | 0.016 | -0.000 | 0.033 | 0.005 | -0.008 | 0.019 |
| Kidney/liver disease x Age 85+ | 0.018 | -0.006 | 0.043 | -0.005 | -0.026 | 0.015 |
| Long term back problem x Age 65-74 | 0.021 | 0.014 | 0.028 | 0.007 | 0.002 | 0.013 |
| Long term back problem x Age 75-84 | 0.040 | 0.033 | 0.048 | 0.010 | 0.003 | 0.017 |
| Long term back problem x Age 85+ | 0.084 | 0.072 | 0.096 | 0.041 | 0.029 | 0.052 |
| Long term mental health prob. x Age 65-74 | 0.040 | 0.023 | 0.056 | 0.026 | 0.015 | 0.037 |
| Long term mental health prob. x Age 75-84 | 0.029 | 0.006 | 0.052 | 0.020 | 0.003 | 0.036 |
| Long term mental health prob. x Age 85+ | 0.017 | -0.025 | 0.060 | -0.010 | -0.045 | 0.024 |
| **Variable** | **Coefficient** | **95% CI**  **Lower, Upper** | | **Coefficient** | **95% CI**  **Lower, Upper** | |
| Long term neurological prob. x Age 65-74 | 0.031 | 0.012 | 0.050 | 0.023 | 0.009 | 0.037 |
| Long term neurological prob. x Age 75-84 | 0.057 | 0.035 | 0.079 | 0.027 | 0.011 | 0.042 |
| Long term neurological prob. x Age 85+ | 0.075 | 0.039 | 0.112 | 0.032 | 0.000 | 0.063 |
| Another long term condition x Age 65-74 | 0.013 | 0.007 | 0.018 | 0.010 | 0.006 | 0.014 |
| Another long term condition x Age 75-84 | 0.003 | -0.004 | 0.010 | 0.002 | -0.003 | 0.007 |
| Another long term condition x Age 85+ | -0.018 | -0.031 | -0.006 | -0.022 | -0.032 | -0.013 |
| Health limited by recent illness or injury | -0.118 | -0.120 | -0.116 | -0.074 | -0.076 | -0.072 |
| Age18-24 | 0.028 | 0.026 | 0.031 | 0.022 | 0.020 | 0.025 |
| Age25-34 | 0.025 | 0.023 | 0.027 | 0.019 | 0.017 | 0.022 |
| Age35-44 | 0.015 | 0.013 | 0.017 | 0.011 | 0.009 | 0.013 |
| Age45-54 | 0.003 | 0.001 | 0.005 | -0.001 | -0.003 | 0.001 |
| Age65-74 | -0.010 | -0.012 | -0.007 | 0.000 | -0.002 | 0.003 |
| Age75-84 | -0.056 | -0.060 | -0.053 | -0.020 | -0.024 | -0.017 |
| Age 85plus | -0.165 | -0.173 | -0.158 | -0.101 | -0.108 | -0.094 |
| Male | 0.006 | 0.005 | 0.007 | 0.001 | 0.000 | 0.002 |
| Mixed | -0.038 | -0.060 | -0.016 | -0.009 | -0.029 | 0.010 |
| Chinese | 0.020 | 0.005 | 0.036 | 0.019 | -0.001 | 0.039 |
| Asian | -0.017 | -0.025 | 0.009 | -0.010 | -0.016 | -0.003 |
| Black African or Caribbean | -0.003 | -0.013 | 0.008 | 0.016 | 0.007 | 0.026 |
| Other including Arab | -0.031 | -0.044 | -0.019 | -0.009 | -0.017 | -0.000 |
| IMD | -0.001 | -0.001 | -0.001 | -0.001 | -0.001 | -0.001 |
| Intercept | 0.933 | 0.932 | 0.935 | 0.0943 | 0.941 | 0.944 |
|  |  |  |  |  |  |  |
| Correlation between main equation and selection equation |  |  |  | -0.985 | -0.986 | -0.983 |
| Adjusted R^2^ | 0.464 |  |  |  |  |  |
| Root MSE | 0.188 |  |  |  |  |  |
| Wald Chi2(176) |  |  |  | 193666; p<0.0001 | | |

MH: indicator for mental health condition; * Model included medical conditions-age interactions for age 18-24, 25-34, 35-44 and 45 to 54 indicators and interactions for two age indicators, age 65 years and older and age under 55, with ethnicity indicators (so that coefficients on ethnicity represent age 55-64 ethnic groups’ utility difference relative to the reference group: White, female, age 55-64 at mean multiple deprivation level, no long term conditions and no recent health limitation or injury affecting health on the day of survey). IMD: index of multiple deprivation as a deviation from the mean for the overall sample. Binary indicator controls for missing age, gender and ethnicity were also included. ** Bivariate model of normal and probit equations, estimated by maximum likelihood. Censored observations: N=58713; Uncernsored observations: 854786.

The adjusted estimates of utility differences between individuals with physical multi-morbidities and individuals without them are calculated by adding the indicator coefficient for the number of conditions and the estimated coefficient for the condition-specific indicators. For example, from figures on the second column of the table, the utility of individuals reporting diabetes, high blood pressure and no other condition is calculated as the sum of the respective coefficients specific to these conditions (-0.060 – 0.022=-0.082) plus the estimated coefficient of the indicator for having a total of two conditions (‘Indicator 2 conditions’; i.e. 0.006). This means that the average impact of two condition multimorbidity is lower than the sum of the effects of the respective single morbidity effects (i.e. the effect is sub-additive: multimorbidity impact is -0.076 vs. -0.082). For three condition multimorbidity the sum of the single effects is not different from their combined effect as multimorbidity (i.e. coefficient of ‘Indicator 3 conditions’ is -0.004 (95% CI: -0.009, 0.001)). With four or more conditions the effect of multimorbidity is super-additive or synergistic as it is larger than the sum of the effects of the conditions when occurring alone by -0.012 (95% CI: -0.020, -0.003; see ‘Indicator 4 or more conditions’). In cases where long term mental problems are present, the interactions of mental problems and number of non-mental multimorbidities (i.e. ‘Indicator 2 conditions x MH’, ‘Indicator 3 conditions x MH’ and ‘Indicator 4 or more conditions x MH’) make the three levels of non-mental multimorbidity synergistic.

Also of main interest are the interactions of specific conditions with age 65-74, 75-84 and 85+ age. These estimates show that the impact of single morbidity is lower in the elderly (aged 65 and older) than in younger (55-64) individuals, consistent with findings on multimorbidity effects across young and old reported in the main body of the text.

The second, third and fourth column present the results for the main, ‘reductionist’, model. The last three columns of the table present the results of a sensitivity analysis adjusting for item non-response; this analysis consisted in simultaneously estimating the same EQ-5D index score equation as in the original model and a selection equation having as dependent variable a binary indicator taking the value of 1 if the respondent had a recorded EQ-5D score or 0 if he did not (i.e. item non-response). The selection or item non response equation is a function of the same covariates as the main equation (with the exception of age by long term condition interactions, the age by ethnicity interactions, and the variable indicating health limitations due to a recent condition or illness), plus an instrumental variable, which we arbitrarily chose to be that of reported sexual orientation, dichotomised between ‘heterosexual/straight’ equalling 1 and 0 for ‘gay/lesbian’, ‘other’ or ‘prefer not to say’ (see Question 57 in GGPS 2011-12). The model assumes that the error terms for the EQ-5D index equation, and the selection equation (the results of which are not presented in the table), are jointly distributed as bivariate normal, with mean zero for both terms, a variance of 1 for the selection equation, and an EQ-5D error variance and covariance with the selection equation error to be estimated. If different from zero, the last term would serve to account for the effect of unobserved confounding on EQ-5D scores. The selection model was estimated by maximum likelihood methods, using a truncated normal density for EQ-5D scores and a cumulative normal distribution (probit) for the selection equation^[[1]](#endnote-1)^.

The selection probit model included data on 854,786 respondents; 58,713 were for EQ-5D non-respondents; 796,073 were for those providing EQ-5D data. Its Wald chi2 test statistic (98 degrees of freedom) was 12123.70 (p = 0.000). The correlation estimate was found to be equal to -0.98 and highly significant (p<0.0001), suggesting informative item non-response. The coefficient estimate for the variable indicator of not stating heterosexual/straight in Q57, had a negative coefficient with a z statistic of 7.1 (not shown); this suggests that those who stated something other than ‘heterosexual/straight’ were less likely to provide EQ-5D index score data.

These results suggest that a) item non-response is non-ignorable, and b) those who , given their observed characteristics, have higher than average probability of providing EQ-5D score data have lower than average EQ-5D scores. However, the results of the model that adjusted for non-ignorable item non-response are generally not significantly different in clinical terms from those of the fixed effects model, which assumes ignorable non-response. Only the estimated coefficients for interactions of age and long term back problems, age and arthritis, and mental health and back problems differ between the two models by more than 0.03 points. These results give some support for the fixed effects model results presented in the main paper. Further research is warranted to verify that results to the fixed effects model remain robust to adjustment for item non-response in large samples collecting more background characteristics of respondents, such as education, marital status, household size and composition, which permit testing for various specifications of the selection equation.

Appendix Table A5: Interactions between two and three conditions (Model 2)

| **Conditions** | **Association with full health-equivalent days per year** | **Lower 95% CI** | **Upper 95% CI** |
| --- | --- | --- | --- |
| Single conditions | | | |
| Angina/heart | -25 | -28 | -23 |
| Arthritis/joint | -70 | -72 | -69 |
| Asthma/chest | -16 | -18 | -14 |
| Cancer | -40 | -43 | -38 |
| Deafness/severe hearing impairment | -17 | -20 | -13 |
| Diabetes | -13 | -15 | -11 |
| Epilepsy | -10 | -16 | -4 |
| High blood pressure | -7 | -8 | -6 |
| Kidney/liver | -34 | -39 | -28 |
| Long term back problem | -64 | -66 | -61 |
| Mental health problem | -78 | -82 | -74 |
| Neurological | -99 | -105 | -93 |
| Combinations of three conditions (n=220 combinations)  (Model 2 with dyads & triads vs. dyads only F=19.3 (286, 823121), p<0.0001) | | | |
| triad:Asthma_Arthritis_Angina | -1 | -12 | 10 |
| triad:Cancer_Arthritis_Angina | 24 | 7 | 40 |
| triad:Cancer_Asthma_Angina | 5 | -22 | 32 |
| triad:Cancer_Asthma_Arthritis | 15 | -2 | 31 |
| triad:Deaf_Arthritis_Angina | -14 | -25 | -2 |
| triad:Deaf_Asthma_Angina** | -46 | -69 | -24 |
| triad:Deaf_Asthma_Arthritis | -7 | -19 | 5 |
| triad:Deaf_Cancer_Angina | -3 | -23 | 16 |
| triad:Deaf_Cancer_Arthritis | 17 | 1 | 33 |
| triad:Deaf_Cancer_Asthma | -8 | -35 | 19 |
| triad:Diabetes_Arthritis_Angina | -11 | -23 | 1 |
| triad:Diabetes_Asthma_Angina** | -31 | -47 | -16 |
| **Conditions** | **Association with full health-equivalent days per year** | **Lower 95% CI** | **Upper 95% CI** |
| Combinations of three conditions (n=220 combinations)  (Model 2 with dyads & triads vs. dyads only F=19.3 (286, 823121), p<0.0001) | | | |
| triad:Diabetes_Asthma_Arthritis** | -25 | -39 | -11 |
| triad:Diabetes_Cancer_Angina | -31 | -62 | 1 |
| triad:Diabetes_Cancer_Arthritis | 0 | -19 | 19 |
| triad:Diabetes_Cancer_Asthma | -19 | -57 | 19 |
| triad:Diabetes_Deaf_Angina** | -31 | -49 | -13 |
| triad:Diabetes_Deaf_Arthritis** | -32 | -49 | -15 |
| triad:Diabetes_Deaf_Asthma | -20 | -40 | 1 |
| triad:Diabetes_Deaf_Cancer | -1 | -22 | 21 |
| triad:Epilepsy_Arthritis_Angina | -15 | -56 | 26 |
| triad:Epilepsy_Asthma_Angina** ~ | -57 | -102 | -12 |
| triad:Epilepsy_Asthma_Arthritis | -23 | -63 | 17 |
| triad:Epilepsy_Cancer_Angina | -34 | -119 | 52 |
| triad:Epilepsy_Cancer_Arthritis | -32 | -77 | 12 |
| triad:Epilepsy_Cancer_Asthma | 11 | -48 | 70 |
| triad:Epilepsy_Deaf_Angina | -48 | -110 | 15 |
| triad:Epilepsy_Deaf_Arthritis | -11 | -39 | 17 |
| triad:Epilepsy_Deaf_Asthma | -12 | -70 | 45 |
| triad:Epilepsy_Deaf_Cancer | -101 | -252 | 50 |
| triad:Epilepsy_Diabetes_Angina | -32 | -79 | 15 |
| triad:Epilepsy_Diabetes_Arthritis | -67 | -153 | 18 |
| triad:Epilepsy_Diabetes_Asthma | 15 | -26 | 55 |
| triad:Epilepsy_Diabetes_Cancer | -125 | -272 | 22 |
| triad:Epilepsy_Diabetes_Deaf | -6 | -67 | 54 |
| triad:HBP_Arthritis_Angina | -10 | -15 | -4 |
| triad:HBP_Asthma_Angina** | -22 | -30 | -14 |
| triad:HBP_Asthma_Arthritis | -14 | -19 | -8 |
| triad:HBP_Cancer_Angina | 4 | -8 | 15 |
| triad:HBP_Cancer_Arthritis | 10 | 3 | 17 |
| triad:HBP_Cancer_Asthma | 9 | -2 | 21 |
| triad:HBP_Deaf_Angina** | -22 | -29 | -15 |
| triad:HBP_Deaf_Arthritis | -16 | -23 | -10 |
| triad:HBP_Deaf_Asthma** | -22 | -32 | -13 |
| **Conditions** | **Association with full health-equivalent days per year** | **Lower 95% CI** | **Upper 95% CI** |
| Combinations of three conditions (n=220 combinations)  (Model 2 with dyads & triads vs. dyads only F=19.3 (286, 823121), p<0.0001) | | | |
| triad:HBP_Deaf_Cancer | -8 | -20 | 3 |
| triad:HBP_Diabetes_Angina** | -18 | -25 | -12 |
| triad:HBP_Diabetes_Arthritis** | -19 | -24 | -15 |
| triad:HBP_Diabetes_Asthma | -11 | -17 | -5 |
| triad:HBP_Diabetes_Cancer | 9 | 1 | 16 |
| triad:HBP_Diabetes_Deaf** | -19 | -28 | -11 |
| triad:HBP_Epilepsy_Angina | -15 | -37 | 7 |
| triad:HBP_Epilepsy_Arthritis | -4 | -23 | 16 |
| triad:HBP_Epilepsy_Asthma | 1 | -30 | 31 |
| triad:HBP_Epilepsy_Cancer | -13 | -56 | 29 |
| triad:HBP_Epilepsy_Deaf | 17 | -27 | 60 |
| triad:HBP_Epilepsy_Diabetes | -2 | -25 | 21 |
| triad:Kidney/liver_Arthritis_Angina | 4 | -23 | 31 |
| triad:Kidney/liver_Asthma_Angina** | -65 | -116 | -14 |
| triad:Kidney/liver_Asthma_Arthritis | -9 | -40 | 22 |
| triad:Kidney/liver_Cancer_Angina | 29 | -17 | 76 |
| triad:Kidney/liver_Cancer_Arthritis | 39 | -4 | 82 |
| triad:Kidney/liver_Cancer_Asthma** | -83 | -138 | -28 |
| triad:Kidney/liver_Deaf_Angina | -65 | -134 | 4 |
| triad:Kidney/liver_Deaf_Arthritis | -40 | -79 | -1 |
| triad:Kidney/liver_Deaf_Asthma | -7 | -46 | 32 |
| triad:Kidney/liver_Deaf_Cancer | -35 | -107 | 38 |
| triad:Kidney/liver_Diabetes_Angina | -15 | -39 | 9 |
| triad:Kidney/liver_Diabetes_Arthritis | -31 | -68 | 6 |
| triad:Kidney/liver_Diabetes_Asthma** | -71 | -110 | -32 |
| triad:Kidney/liver_Diabetes_Cancer | 5 | -34 | 44 |
| triad:Kidney/liver_Diabetes_Deaf | 1 | -50 | 51 |
| triad:Kidney/liver_Epilepsy_Angina | 19 | -67 | 105 |
| triad:Kidney/liver_Epilepsy_Arthritis | -5 | -127 | 117 |
| triad:Kidney/liver_Epilepsy_Asthma ~ | -60 | -117 | -4 |
| triad:Kidney/liver_Epilepsy_Cancer^^ ~ | 138 | 114 | 163 |
| triad:Kidney/liver_Epilepsy_Deaf** ~ | -149 | -168 | -130 |
| **Conditions** | **Association with full health-equivalent days per year** | **Lower 95% CI** | **Upper 95% CI** |
| Combinations of three conditions (n=220 combinations)  (Model 2 with dyads & triads vs. dyads only F=19.3 (286, 823121), p<0.0001) | | | |
| triad:Kidney/liver_Epilepsy_Diabetes | -38 | -172 | 96 |
| triad:Kidney/liver_HBP_Angina | -8 | -23 | 7 |
| triad:Kidney/liver_HBP_Arthritis | 11 | -3 | 24 |
| triad:Kidney/liver_HBP_Asthma | -17 | -35 | 1 |
| triad:Kidney/liver_HBP_Cancer | 9 | -9 | 27 |
| triad:Kidney/liver_HBP_Deaf | -2 | -23 | 18 |
| triad:Kidney/liver_HBP_Diabetes | -16 | -29 | -3 |
| triad:Kidney/liver_HBP_Epilepsy | -2 | -28 | 23 |
| triad:Back_Arthritis_Angina | 19 | 10 | 28 |
| triad:Back_Asthma_Angina | 6 | -15 | 27 |
| triad:Back_Asthma_Arthritis | -1 | -8 | 7 |
| triad:Back_Cancer_Angina | 32 | 5 | 59 |
| triad:Back_Cancer_Arthritis^^ | 34 | 21 | 47 |
| triad:Back_Cancer_Asthma | 27 | 3 | 51 |
| triad:Back_Deaf_Angina | -14 | -37 | 8 |
| triad:Back_Deaf_Arthritis | 15 | 5 | 24 |
| triad:Back_Deaf_Asthma | -15 | -34 | 4 |
| triad:Back_Deaf_Cancer | 1 | -30 | 33 |
| triad:Back_Diabetes_Angina | -39 | -69 | -8 |
| triad:Back_Diabetes_Arthritis | -5 | -17 | 6 |
| triad:Back_Diabetes_Asthma | -31 | -52 | -9 |
| triad:Back_Diabetes_Cancer | -15 | -62 | 32 |
| triad:Back_Diabetes_Deaf | -34 | -63 | -4 |
| triad:Back_Epilepsy_Angina** ~ | -71 | -121 | -21 |
| triad:Back_Epilepsy_Arthritis | -5 | -35 | 25 |
| triad:Back_Epilepsy_Asthma** | -144 | -226 | -63 |
| triad:Back_Epilepsy_Cancer | -34 | -165 | 98 |
| triad:Back_Epilepsy_Deaf | -32 | -112 | 47 |
| triad:Back_Epilepsy_Diabetes | -55 | -162 | 52 |
| triad:Back_HBP_Angina | -11 | -21 | -2 |
| triad:Back_HBP_Arthritis | 6 | 2 | 11 |
| triad:Back_HBP_Asthma | -6 | -15 | 2 |
| **Conditions** | **Association with full health-equivalent days per year** | **Lower 95% CI** | **Upper 95% CI** |
| Combinations of three conditions (n=220 combinations)  (Model 2 with dyads & triads vs. dyads only F=19.3 (286, 823121), p<0.0001) | | | |
| triad:Back_HBP_Cancer | 7 | -11 | 26 |
| triad:Back_HBP_Deaf | -7 | -19 | 5 |
| triad:Back_HBP_Diabetes** | -24 | -32 | -16 |
| triad:Back_HBP_Epilepsy | -39 | -105 | 27 |
| triad:Back_Kidney/liver_Angina** | -92 | -170 | -14 |
| triad:Back_Kidney/liver_Arthritis | 31 | 7 | 55 |
| triad:Back_Kidney/liver_Asthma | -13 | -89 | 63 |
| triad:Back_Kidney/liver_Cancer | 49 | 7 | 92 |
| triad:Back_Kidney/liver_Deaf** ~ | -126 | -185 | -68 |
| triad:Back_Kidney/liver_Diabetes | -17 | -57 | 22 |
| triad:Back_Kidney/liver_Epilepsy | 21 | -6 | 48 |
| triad:Back_Kidney/liver_HBP | 16 | -2 | 34 |
| triad:MH_Arthritis_Angina | -19 | -53 | 15 |
| triad:MH_Asthma_Angina | -6 | -48 | 37 |
| triad:MH_Asthma_Arthritis | -27 | -47 | -7 |
| triad:MH_Cancer_Angina | -39 | -130 | 52 |
| triad:MH_Cancer_Arthritis | -16 | -59 | 27 |
| triad:MH_Cancer_Asthma | -45 | -103 | 13 |
| triad:MH_Deaf_Angina | -60 | -125 | 5 |
| triad:MH_Deaf_Arthritis | -24 | -59 | 12 |
| triad:MH_Deaf_Asthma | 18 | -24 | 61 |
| triad:MH_Deaf_Cancer | 15 | -29 | 59 |
| triad:MH_Diabetes_Angina | -27 | -89 | 35 |
| triad:MH_Diabetes_Arthritis | 3 | -26 | 32 |
| triad:MH_Diabetes_Asthma** | -50 | -82 | -18 |
| triad:MH_Diabetes_Cancer | 1 | -48 | 51 |
| triad:MH_Diabetes_Deaf | -32 | -89 | 26 |
| triad:MH_Epilepsy_Angina | 16 | -15 | 47 |
| triad:MH_Epilepsy_Arthritis | -41 | -117 | 34 |
| triad:MH_Epilepsy_Asthma | -65 | -142 | 11 |
| triad:MH_Epilepsy_Cancer | -19 | -210 | 172 |
| triad:MH_Epilepsy_Deaf | -10 | -102 | 81 |
| **Conditions** | **Association with full health-equivalent days lost per year** | **Lower 95% CI** | **Upper 95% CI** |
| Combinations of three conditions (n=220 combinations)  (Model 2 with dyads & triads vs. dyads only F=19.3 (286, 823121), p<0.0001) | | | |
| triad:MH_Epilepsy_Diabetes** ~ | -100 | -150 | -51 |
| triad:MH_HBP_Angina | -23 | -51 | 5 |
| triad:MH_HBP_Arthritis | -14 | -30 | 1 |
| triad:MH_HBP_Asthma | -3 | -20 | 15 |
| triad:MH_HBP_Cancer | -14 | -51 | 24 |
| triad:MH_HBP_Deaf | 22 | -3 | 47 |
| triad:MH_HBP_Diabetes | -23 | -42 | -4 |
| triad:MH_HBP_Epilepsy | -89 | -179 | 2 |
| triad:MH_Kidney/liver_Angina~ | 85 | 8 | 163 |
| triad:MH_Kidney/liver_Arthritis | 48 | -8 | 104 |
| triad:MH_Kidney/liver_Asthma** | -95 | -157 | -32 |
| triad:MH_Kidney/liver_Cancer** ~ | -98 | -164 | -31 |
| triad:MH_Kidney/liver_Deaf | -120 | -254 | 13 |
| triad:MH_Kidney/liver_Diabetes | -7 | -69 | 56 |
| triad:MH_Kidney/liver_Epilepsy**~ | -102 | -162 | -41 |
| triad:MH_Kidney/liver_HBP | -15 | -56 | 26 |
| triad:MH_Back_Angina | 30 | -15 | 75 |
| triad:MH_Back_Arthritis | -19 | -35 | -4 |
| triad:MH_Back_Asthma | -20 | -40 | 0 |
| triad:MH_Back_Cancer | -46 | -113 | 21 |
| triad:MH_Back_Deaf | -50 | -97 | -3 |
| triad:MH_Back_Diabetes | -1 | -32 | 30 |
| triad:MH_Back_Epilepsy | -30 | -105 | 45 |
| triad:MH_Back_HBP | -25 | -45 | -5 |
| triad:MH_Back_Kidney/liver | -19 | -77 | 39 |
| triad:Neurological_Arthritis_Angina | 38 | -12 | 88 |
| triad:Neurological_Asthma_Angina | 16 | -33 | 65 |
| triad:Neurological_Asthma_Arthritis | 33 | 2 | 63 |
| triad:Neurological_Cancer_Angina | 48 | -43 | 140 |
| triad:Neurological_Cancer_Arthritis^^ | 84 | 30 | 138 |
| triad:Neurological_Cancer_Asthma | 33 | -44 | 111 |
| triad:Neurological_Deaf_Angina^^ ~ | 72 | 45 | 99 |
| **Conditions** | **Association with full health-equivalent days per year** | **Lower 95% CI** | **Upper 95% CI** |
| Combinations of three conditions (n=220 combinations)  (Model 2 with dyads & triads vs. dyads only F=19.3 (286, 823121), p<0.0001) | | | |
| triad:Neurological_Deaf_Arthritis | 56 | 1 | 112 |
| triad:Neurological_Deaf_Asthma | 23 | -35 | 81 |
| triad:Neurological_Deaf_Cancer | 60 | -20 | 140 |
| triad:Neurological_Diabetes_Angina | 48 | 4 | 92 |
| triad:Neurological_Diabetes_Arthritis | 0 | -42 | 41 |
| triad:Neurological_Diabetes_Asthma | -27 | -95 | 41 |
| triad:Neurological_Diabetes_Cancer | -31 | -124 | 61 |
| triad:Neurological_Diabetes_Deaf | 15 | -106 | 135 |
| triad:Neurological_Epilepsy_Angina | 30 | -51 | 111 |
| triad:Neurological_Epilepsy_Arthritis | 65 | -16 | 146 |
| triad:Neurological_Epilepsy_Asthma | 28 | -28 | 85 |
| triad:Neurological_Epilepsy_Cancer | 37 | -35 | 109 |
| triad:Neurological_Epilepsy_Deaf | 3 | -77 | 83 |
| triad:Neurological_Epilepsy_Diabetes | -28 | -111 | 55 |
| triad:Neurological_HBP_Angina | 19 | -8 | 47 |
| triad:Neurological_HBP_Arthritis^^ | 43 | 27 | 59 |
| triad:Neurological_HBP_Asthma | 38 | 2 | 75 |
| triad:Neurological_HBP_Cancer | 26 | -18 | 70 |
| triad:Neurological_HBP_Deaf | 29 | -1 | 59 |
| triad:Neurological_HBP_Diabetes | 4 | -17 | 26 |
| triad:Neurological_HBP_Epilepsy | -9 | -63 | 44 |
| triad:Neurological_Kidney/liver_Angina | 58 | -60 | 176 |
| triad:Neurological_Kidney/liver_Arthritis | 81 | -9 | 171 |
| triad:Neurological_Kidney/liver_Asthma~ | 76 | 6 | 145 |
| triad:Neurological_Kidney/liver_Cancer^^~ | 86 | 31 | 141 |
| triad:Neurological_Kidney/liver_Deaf | -134 | -317 | 50 |
| triad:Neurological_Kidney/liver_Diabetes | 25 | -7 | 56 |
| triad:Neurological_Kidney/liver_Epilepsy | 46 | -89 | 182 |
| triad:Neurological_Kidney/liver_HBP | 15 | -27 | 58 |
| triad:Neurological_Back_Angina | 26 | -39 | 91 |
| triad:Neurological_Back_Arthritis^^ | 50 | 35 | 65 |
| triad:Neurological_Back_Asthma | 16 | -16 | 48 |
| **Conditions** | **Association with full health-equivalent days per year** | **Lower 95% CI** | **Upper 95% CI** |
| Combinations of three conditions (n=220 combinations)  (Model 2 with dyads & triads vs. dyads only F=19.3 (286, 823121), p<0.0001) | | | |
| triad:Neurological_Back_Cancer^^ | 84 | 32 | 136 |
| triad:Neurological_Back_Deaf^^ | 59 | 19 | 98 |
| triad:Neurological_Back_Diabetes | -34 | -83 | 15 |
| triad:Neurological_Back_Epilepsy | -11 | -62 | 41 |
| triad:Neurological_Back_HBP | 29 | 8 | 49 |
| triad:Neurological_Back_Kidney/liver | 76 | -15 | 167 |
| triad:Neurological_MH_Angina | 4 | -140 | 149 |
| triad:Neurological_MH_Arthritis | -7 | -47 | 33 |
| triad:Neurological_MH_Asthma | 35 | -15 | 84 |
| triad:Neurological_MH_Cancer | 67 | -3 | 137 |
| triad:Neurological_MH_Deaf | -51 | -134 | 32 |
| triad:Neurological_MH_Diabetes^^ ~ | 85 | 21 | 149 |
| triad:Neurological_MH_Epilepsy | -1 | -59 | 56 |
| triad:Neurological_MH_HBP | -3 | -46 | 40 |
| triad:Neurological_MH_Kidney/liver** ~ | -111 | -209 | -13 |
| triad:Neurological_MH_Back | -5 | -40 | 29 |

Rows corresponding to conditions with super-additive effects(i.e. interaction term was negative) are highlighted in grey. Combinations of conditions with sub-additive effects (interaction term was positive) are highlighted in blue. Other combinations of conditions are those with additive effects (i.e. interaction terms statistically insignificantly different from zero).

** Super-additive effect that is significantly different from zero and clinically significant (Upper 95% CI bound is less than -11).

^^ Sub-additive effect that is significantly different from zero and clinically significant (Lower 95% CI bound is higher than 11)

~ Combinations with fewer than 20 observations.

1. Heckman JJ. Dummy Endogenous Variables in a Simultaneous Equation System. *Econometrica 1978*, 46, 931–959 [↑](#endnote-ref-1)
